# Supplementary material for: Lived experiences of frontline healthcare providers offering maternal and newborn services amidst the novel corona virus disease 19 pandemic in Uganda: A qualitative study
Source: PLoS One. 2021 Dec 10;16(12):e0259835. doi: 10.1371/journal.pone.0259835 (PMC8664167; doi:10.1371/journal.pone.0259835)
Supplement: S1 Dataset — (DOCX) [file pone.0259835.s001.docx]

1. **Experiences and perceptions**

**Experience of service before pandemic**

**Busy days dedicated to specialist services**

During COVID-19 lockdown, there were days that were designated for specialised services. This means that the routine was altered and had to improvise a way of attending to patients that had specialised needs. This however differed from hospital to hospital as well as departments. Therefore, the normal routine of the hospital changed as a result of the situation at that time.

*We have always had a busy schedule and my schedule is from Monday to Saturday. Monday, Tuesday, Thursday, and Friday, I am not always very busy but Wednesday and Saturday are always very busy. Since the hospital started with Wednesday and Saturday as immunisation days, so they have constantly been busy.* **Obstetrician, Public hospital**

**Adequate staffing**

Participants also revealed that during the lockdown and COVID-19, there were always enough staff at the hospital to attend to the patients. All the health worker categories were available and work was going on as usual. So if there was any complication, it would easily be handled because of the fact that all the health workers were there as the following excerpts suggest.

*We had enough staff and support staff and everything was normal as the ordinary. There was not much. Of course, everything was going on as expected.* **Midwife, Private-Not-for Profit hospital**

*Before COVID-19, work has been going so well because we have around six gynaecologists. You know when you work around with big doctors you get different ideas, you are able to improve your skills, you can ask questions and they able to explain and teach you how you are supposed to do certain procedures. You learn a lot from them.* **Midwife, Private-Not-for Profit hospital**

**Steady patient in-flows**

Some of the participants reported that there was a steady inflow of patients in as much as the lockdown was there. Somehow, the hospitals kept receiving patients from all lifestyles and therefore needed services to be continued. It is important to note however that the numbers of the patients relatively reduced from what they were seeing before COVID-19.

*Actually before COVID-19 we had the numbers and the flow was actually known. We used to have like 250 deliveries a month and you would know that was the average but when COVID-19 came in, the numbers reduced significantly and I wonder where people were giving birth* **Midwife, Private-Not-for Profit hospital**

*Before the COVID-19 pandemic, you can see that our numbers were higher and we were generating enough revenue but with COVID-19, the lockdown and curfew, our numbers dropped.* **Obstetrician, Private-Not-for Profit hospital**

**Quick and adequate supply of essential commodities**

Some of the participants also reported that there was a steady supply of medical equipment. Everything that the health workers needed was received in real time. This seemed to be rather a surprise as they thought that this would change and run into shortages.

*Before COVID-19, everything was running on very well, the supply of the medicine and the sundries was running very quickly, everything we had it in time.* **Midwife, Private hospital**

**Working with colleagues in teams**

Importantly, before the period of lockdown and the pandemic, the health workers were working in harmony and everything was done as a team. This meant that some of the health workers were empowered to do some procedures that they would have otherwise not done if it was not for the pandemic.

*Well before COVID-19, you could report on duty and you would be very sure to expect to find at least one colleague there. You would examine the patient, ask all your questions, hand over the patient physically, see and manage the gaps in what you have recorded, call back the mother and even ask further questions because the mother was present and not worried about going home and what time they will leave.* **Obstetrician, Private-Not-for Profit hospital**

**Perceptions on maternal and new born health care during the pandemic**

**Passion and dedication to serve**

Participants also reported the love for work as one of the important aspects of the health workers role. This is because the happiness of bring a new life as well saving a mother from danger was one of the important sentiments that the participants held. With this, whether in lockdown or emergency considerations, there should be always somebody to help. This was due to the premise that labour is not predetermined and comes at any minute without warning.

*You know with maternity and delivery, its about passion – you have to have the heart to do it as a person. You are dealing with two people and both of them want to be alive. That alone can motivate you to serve. Yes because we are saving life and bring new life all together. When you have a child you are reminded that you were also helped so you have a child to date. I love the profession and sometimes I think I was actually born and predestined to be a nurse and midwife [laughs].* **Midwife, Private hospital**

Tireless efforts to avoid complications or mishaps

Maternal and new-born health is such a critical consideration because securing child birth, anything can happen. All these processes lie at the back of the health workers and therefore a need for them (health workers) to work hard and always be on duty especially those at the forefront of this urgent and rather noble process of bringing a new life on earth as the following excerpt reveals.

*We really needed to work our best to make sure that we don’t get any problem. Because you know sometimes anything can happen, though it… anything can happen and you can be sued, they can take the information to the administration and I think somehow you are put on the disciplinary committee.* **Obstetrician, Private-Not-for Profit hospital**

Having a healthy mother and healthy baby in the midst of COVID-19

Similarly, having a health and bouncing baby also played a part in ensuring that the maternal and new born health services remained essential. Essential in a way that even the health workers especially those called to work found it easy to move amidst their different destinations to attend to these mothers. Some of the health workers reported their passion to see a health bunch of a mother and baby in a good health after birth. Besides, who else would do the job is they were not able to attend to it. The following quote exemplifies the described sentiments.

*I love to see that am bringing new life to the earth. A healthy mother and a healthy baby that is my passion. I Love doing it without even being pushed. Yesterday, I was here at 2am in the night, they called me to attend to a mother, I got into the car and drove all the 10 miles to get here in the darkness with all the fears. It takes me about 40 minutes to get here. For me I just love seeing the mother, have a healthy baby and the mother is safe.* **Obstetrician, Private hospital**

**Changes during the pandemic/ How did the lockdown affect you**

**Working on specific days only**

One of the changes that were experienced because of COVID-19 and the consequent lockdown the realisation that most of the health workers were not going to work full time. This was because of the fact that the clients were few in some departments. Some of the health workers were supposed to work on the designated to work on specific days and not all the time as they used to do before COVID-19 and the lockdown respectively. In some health facilities, arrangements were made to pick up health workers who were on duty.

*It changed because when the pandemic began, the hospital made an arrangement of going back to the usual days of immunisation it started with. That is Wednesday and Saturday because of transport issues. So the hospital made arrangements for my transportation for only two days. So I would only come here on Wednesday and Saturday.* **Midwife, Private hospital**

*Also time was an issue because the curfew was 6:00am up to 7:00pm so by 3:00pm people were leaving and going back. So you have few left and the work overload shoots up.* **Midwife, Public hospital**

**Closure of services provided**

Due to COVID-19, some facilities had to close off some of the services and resorted to those that were more critical. This was to make sure that people are safe. Participants shared that some of the services that were put on a halt during this time were activities related to outreach services. This was because of transport issues as well as reducing crowds and really had to improvise on how to deliver some of these services especially for immunisation.

*Of course when the lockdown came, some of the services were closed and so as Head of department especially because it is special or much needed service, you would negotiate with management on how to best provide the service in a safe way* **Obstetrician, Private hospital**

**Limited working times**

Because of curfew, some people had to work less time than they used to work. This is because they had to make time to go home before curfew time, which would start at 7Pm and for the motorcycles, at 2Pm. Therefore, this was very challenging as health workers were always in a rush to work on the available people very fast to make time to get home early. This was mainly not to be at logger heads with the police and other security operatives that were enforcing curfew. And in most cases, it was very difficult to beat curfew since it was in the early hours of the evening and also depended on the day.

*Difficulties of meeting curfew times Curfew time; as they were taking us back home, police officers would stop us and on each roadblock, you would have to show your ID. And of course, you would worry if you would get home safely* **Midwife, Private-Not-for Profit hospital**

*Curfew time; as they were taking us back home, police officers would stop us and on each roadblock, you would have to show your ID. And of course, you would worry if you would get home safely* **Midwife, Public hospital**

**Staff-cuts and laying off workers**

In some of the health facilities especially private facilities, some of the health workers were laid off as business was not forthcoming. So for a team that was full found itself missing some members thus making the work more than they expected during COVID-19 and the lock down.

*Ok the other thing is the staffing reduced to half even on my team. I normally have a team of 22-24 staff but now you have a team of let us say 10 staff. This came in because of the transport issue as many staff were coming from afar. The hospital also tried in those times to provide transport but it became difficult as many were coming from far and widely spread.* **Midwife, Private-Not-for Profit hospital**

**Experiences after Lock down**

**Increase in number of working days to contain the numbers**

Some of the services that were offered on specific days reached appoint and the days designated for it were not enough. Therefore, extra days had to be added to contain the high number of patients that were not being served in those days. For example, if a service was offered three days a week like immunisation, they had either to make it daily or add another.

*The number of days increased and changed before the lockdown was lifted because of the overwhelming numbers on Wednesday and Saturday, we had to work extra days.* **Midwife, Private hospital**

**Unaffordable cost of vaccines**

Some of the participants reported that during the Lockdown, some of the items they were using and accessing before, they were not able to get them. This was however on some specific vaccines but not all. This meant that during the COVID-19 lockdown, such vaccinations were not done and thus one wonders what people that were in need of these services.

*During COVID-19, with me here in immunisation, there are vaccines that are supposed to be bought like Hepatitis A, typhoid, MMR and so many others but often, patients would no longer afford because they would tell you that we can no longer afford. So it became very hard for them to buy because most of their businesses had closed.* **Midwife, Private hospital**

**Increase in staff numbers that made work lighter**

In some instances, the participants reported having had an increase in the number of staff that made their work easy. Since there was limited movements, the health workers would come together and move as a group. Similarly, at the facility all medical cadres would be available and therefore consultation was easy as well as referral.

*When they eased vehicles then we had some more staff coming in because they could easily be dropped off and picked using private cars yet previously even the private cars were not moving so when they eased the lockdown some staff had husbands or people who could drop them off and pick them up. Then the number of staff increased a little so transport was and still is a big challenge.* **Midwife, Private-Not-for Profit hospital**

*Return of health care workers, reduced workload, shared maternal care responsibilities.* **Midwife, Public hospital**

**Learnt to be flexible, creative and accommodative of prevailing situations**

It is also important to note that some of the health workers reported having learnt a lesson of being flexible and patient. This is because of all the changes that came with COVID-19 and the lockdown. The situation was so hard that even those who thought that their jobs were everything started thinking twice as this seemed to work only in normal situations.

*I have got a lot of experience and learned to be flexible when things change. When things change, you too have to change. I previously believed that when you have a government job, you are sorted but things changed and during the lockdown, I realised that it is not true. I started to think of other income generating activities I can do to survive because life was difficult, too many responsibilities, people out there believed that as government worker I was still earning during the lockdown so I have money. Yet there was no money at all, I was also depending on salary which I was not 100% sure it was coming.* **Midwife, Public hospital**

**Slight increase in clients attending the facility: Many missing appointments**

In some facilities, some of the participants reported having had an increase in the number of patients more than the days before COVID-19 and the Lockdown. This was because people who would have attended other hospitals were forced to attend to the nearby health facilities. This was more in the government facilities than the private facilities. Equally noting was the fact that many patients missed their appointments due to lack of transport and transport restrictions as the following excerpts justify.

*The number is getting better because now transport is open. However, the transport fare is not so favourable so in the end some still fail to come to the health facility but at least it has improved, it is not bad like it was during the lockdown. Although many miss appointments.* **Midwife, Private-Not-for Profit hospital**

*Turn up of the patients is now raising up but the patients don’t want to put on masks yet we have them in plenty, someone comes with the mask on the chin, you instruct them to put it on properly but in a few minutes its back on the chin. So we are much exposed to being infected by these patients.* **Obstetrician, Private-Not-for Profit hospital**

**Quality of care still not back to expected standards**

Due to COVID-19 and the consequent lockdown, the start of care kind of went down and with the lifting of lockdown, there are still challenges. This means that the quality of services has not yet re-instated as it used to be before the lockdown.

*Also still the quality of care cannot be matched because of the time – things like counselling, information giving. You see mothers who are pregnant need psychological support the most. They must have psychological support and mental support to help them go through their season and come out with a healthy baby. If they are not supported and they are just coming in quickly, get the scan done, get medicines, and then just go away when you are not supporting them so they have a stable mental state so that they have good outcomes at the end of the day then that is not good quality of care.* **Obstetrician, Private hospital**

**Decline in clients’ income, skills and Health facility in financial struggle**

During this time of COVID-19 and the Lockdown, many of the clients to the health facilities lost income and they seeking care was very hard. This was not only on the side of the patients but also on the side of the facilities especially the private facilities as the number of clients greatly declined as the following quotes explain.

*We are currently not offering certain services. This affects our clientele numbers and also our incomes and the skill. If you take long without doing something in medicine, you lose the skill and then that means you need to get some refresher courses so you can come up to speed.* **Midwife, Private-Not-for Profit hospital**

*When you look at the financial perspective, this is a private not for profit hospital and given that the patients numbers had become small during the COVID-19 period, the hospital was affected in terms of income even after the lockdown.* **Midwife, Private hospital**

**Change in operations to fit into COVID-19 SOPs making the service more costly**

Participants went ahead to report that due to COVID-19, services became more expensive as the clients/patients had to incur extra cost. These were costs relating to the cost of medication, masks and other protective equipment that they had to come with to the hospital. Therefore, this is made it hard for some of the people to access health services as the following excerpt reveals.

*We are following the national standard operation guidelines, every person entering examined at the gate, the temperature guns are there, hand sanitizers, when they get to where the doctor is, they are rechecked just to be sure. The services have become a little more expensive on the patient's side. The patient now, as part of the consultation, must pay for a new mask whether or not they have a mask, they pay for a new mask as part of the consultation fee and it is issued as soon as they pay.* **Midwife, Private-Not-for Profit hospital**

**Limit male involvement in antenatal to manage crowding and physical distance**

More still, during this time of COVID-19 and the Lockdown, saw limited involvement of men in antenatal and post-natal services of their wives. This is because few people were allowed to go to the hospital to minimise on overcrowding as well as keeping social distance.

*We had to compromise, you know as obstetricians, we usually want to do antenatal care when the husband is around but now we discourage men from coming to limit the spread of COVID-19 and the congestion. Now in a small consultation room like this one, when the patient is only one, she will sit as far away which creates social distance.* **Obstetrician, Private hospital**

1. **Creation of COVID-19 task force at facility to oversee and manage situations**

There was also creation of the COVID-19 taskforce that was intended to oversee and manage the pandemic. This was a new innovation as it was non-existent because as such a pandemic had never happened. This taskforce was responsible for the

*There is a COVID-19 task force responsible for seeing highest suspicious clients and they are the ones with the PPE's, for us we have the gloves, the masks and the face shields.* **Midwife, Private-Not-for Profit hospital**

**Facilitators, barriers and challenges**

**Facilitators to providing care during the pandemic**

***Passion to serve and creating a safe solution to provide the services***

When participants were asked what facilitated them to continue offering maternal and new born health services, they reported the passion to serve. This was because when the health workers are completing their studies and ready to serve, they swear to save life. Therefore, they found nothing to do other than continuing to offering what they set out to do. To others, they had created a bond with their clients and there had no body to attend to them if they had not done it themselves. Thus the love to serve being one of the most important facilitating factor that kept health workers up and running amidst COVID-19 and Lockdown.

*I am attached to my clients so I had to get a way of managing the situation with the administrators so that I could squeeze time, come to the hospital, and make sure that we offer the services.* **Obstetrician, Private-Not-for Profit hospital**

*I joined the field with passion and asked for this government job with interest. We are told our calling is to love and serve – so I always remembered that motto and it encouraged me and I woke up every morning knowing that this is my calling and I got this government job when I was aware of what was ahead.* **Midwife, Private-Not-for Profit hospital**

*We were also self-motivated [laughs] we had that heart of ‘let me go and serve, because I was called to serve’. I could feel very bad whenever we could see those people coming to the facility, struggling to reach here because there was no transport then spend a whole day here and end up going back home without being attended to.* **Midwife, Private hospital**

*_*

*I love to see that am bringing new life to the earth. A healthy mother and a healthy baby that is my passion. I Love doing it without even being pushed. Yesterday, I was here at 2am in the night, they called me to attend to a mother, I got into the car and drove all the 10 miles to get here in the darkness with all the fears. It takes me about 40 minutes to get here. For me I just love seeing the mother, have a healthy baby and the mother is safe.* **Obstetrician, Private hospital**

**Keeping with the guidelines**

The presence of protective gears and other equipment also made it possible for health workers to continue offering services to their patients. Majority of the participants commended their hospital management for making it possible that everything the health workers needed to protect themselves was made available. This was done in a timely fashion and therefore the health workers had nothing to stop them from coming to work now that they were protected and sure that they may not contract COVID-19 as the following quotes reveal.

*I make sure I am well protected and I do not allow anyone to come in here without putting on a mask. I encourage them to sanitise while coming in. there is a sanitiser right there at the door and to sanitise while going out. As longer as they give me all the protective gears that would protect me, I would be comfortable working on a patient* **Midwife, Public hospital**

*The hospital has done its best to protect us, health workers, it has provided new masks every morning, we have been provided with face shields, we have sanitizers in every consultation room since the index of suspicion is high, we are free to send any suspects for testing as soon as required.* **Midwife, Private-Not-for Profit hospital**

*Interestingly, we did not get any challenges in the medicines, sundries. The hospital did its best to ensure that supplies were available even during the lockdown. They stocked but also we did not have many clients.* **Midwife, Private-Not-for Profit hospital**

**Transport and accommodation support from the hospital**

Some of the participants also reported that the facilities where they worked provided transport services to their staff. This would pick a person from the agreed destination in the morning and drop them off again in the evening. This was a facilitating factor in a way that even during the time when the lockdown was bad and no vehicles were allowed to move, those of the health workers then deemed essential workers were allowed to move. Therefore, the health workers found it easy to come to and from work without fear of clashing with the law. In addition to transport, some of the health facilities tried to create space for accommodation to some health workers to ensure continuity of the services.

*The hospital also tried in those times to provide transport but it became difficult as many were coming from far and widely spread. Organising transport was difficult to reach every destination so those who were staying far were cut off and those staying nearer or could come closer than the buses could pick you up. But there are some people who could not move from where they were to the central bus stage. The hospital also tried to provide nearby accommodation to critical people. Even those who are single and freely accepted to stay around were accommodated but all these were not enough. There are also those who stayed inside the hospital and they provided them with meals* **Midwife, Private-Not-for Profit hospital**

*I stayed here, I was given accommodation here. We have people who stayed here for three months. They were taking us home for one day or once in a while -They drive you home to see your family and they pick you after two days* **Midwife, Private hospital**

*The hospital improvised us with some hostels where some staff were staying and this is those who could not commute from their homes. They also provided cars, which could transport some staff free of charge, and this helped staff who come from far places to make it to work. There are also some organizations which used to give us food for instances staff used to get eggs and posho.* **Midwife, Public hospital**

**Teamwork and support from various stakeholders**

During the lockdown, participants revealed that there was teamwork across all health workers. This is because there was coordination across all section. This made work easy as all the departments were functional and had every health worker in place.

*The advantage here is that we have full support from various stakeholders. We are supported in various ways. We have doctors on duty, we are not working alone. We are a team and if something goes wrong, they are here immediately –the intern or Senior Housing Officer and even a consultant. We get our supplies when we request for them.* **Obstetrician, Public hospital**

*_*

*First of all the corporation we have, the gynaecologists are there and they are good people. You work as a family so when you work as a family, I think you are always happy to come in the morning and say I think am going to work in a place. Am happy and I have peace and go back home well, I think that’s one of the best things we have.* **Midwife, Private-Not-for Profit hospital**

**Remuneration for the service**

The other motivating factor was the fact that some of the health facilities were giving some extra payment for working during COVID-19 times and the Lockdown. Therefore, the health workers felt that they were recognised for their effort to work during COVID-19 and the lockdown. These bonuses were also a welcome gesture to the health workers and an encouragement to help them continue working.

*But you know very well that every job it is a salary. Everybody goes to work to get a salary and its not only in maternity. Even those who sell in hardware shops, policemen, teachers. You see even those who were not working during the lockdown used to get their full pay without a single coin being removed.* **Midwife, Private-Not-for Profit hospital**

*Another motivation from the hospital from the time of lockdown up to today, they are always giving us some bonus. Some little bonus to make us happy every month.* **Midwife, Public hospital**

*Availability of patients needing the service…. The availability of the patients because there is no single day that have come here and have failed to see more than 10 patients. The mothers are always there and it reminds me that the service is needed.* **Midwife, Public hospital**

**The need to earn for a living**

*What motivated me to continue the work during the pandemic is that I had to earn because I have a family to feed so I had to be here.* **Midwife, Private-Not-for Profit hospital**

**Barriers to providing care during the pandemic**

**Staffing challenges and Staff cuts/ shortage in the midst of overwhelming number of patients**

Some of the barriers that the health workers highlighted were related to staff reduction in some of the facilities. This meant that the ones that remained working were over worked and consequently getting burnouts. This was however for a few times during the lockdown and as the lockdown was lifted; there was some relief as the number of health workers that had been stopped from coming started coming back slowly.

*"Sometimes I would get overwhelmed especially during the first two months of the lockdown because I would only come for two days. During those two days, I would really work on so many patients alone. I normally have other people I work with but during the pandemic, most of the staff were reduced.* **Obstetrician, Private-Not-for Profit hospital**

*There was a shortage of labour by then, it was there but we just had to fight it our own way to make things work.* **Midwife, Private-Not-for Profit hospital**

*Sometimes it was hard to run the clinic because you find yourself just one staff attending to so many people and this was overwhelming because of the workload since others failed to come for work.* **Midwife, Public hospital**

*Then also if the numbers are more, you want to beat the numbers and clear the lines so you ensure you review all of them so that no one goes back home without being seen.* **Obstetrician, Private-Not-for Profit hospital**

**Poor time management and restricted movements**

Restricted movements were deemed to be one of the barriers to providing maternal and new born health care at some selected health facilities. This is because there were very many challenges with movements as it was restricted. All those who did not abide by this were either arrested or they were harassed by the law enforcement officers. Therefore, for those who made it to work, worked very fast so that they are not caught up with curfew time.

*The other challenge then was the time management – people had to leave earlier to beat the curfew, then come in late because there was no public transport and they probably walk to work and reach late. It was quite inconveniencing and yet you had staff shortage so there was work overload. But we used to work seriously.* **Midwife, Private-Not-for Profit hospital**

**Inadequate supplies**

It is was worth noting that some facilities did not get enough supplies especially in form of protective gears. This made the work of the health workers hard and thus a barrier to providing maternal and new born health care services in both government, private and not for profit facilities. However, those that were badly hit were the public facilities.

*The challenges were about the supplies to use like the gowns. In maternity you need to have something that covers your body up to the legs but the gowns we have are just these small open gowns yet you really need to close up.* **Midwife, Public hospital**

*One key aspect is the stock out of the major things we need to use like alcohol [alcohol based sanitizer], gloves actually when COVID-19 set in, we lacked all the essentials masks, sanitizers, gloves so it was a big challenge yet very scary. People saying if you have no mask you are not using PPE and yet you are attending to these mothers you are at risk. So it was a big challenge handling the mothers with all the scares* **Midwife, Public hospital**

*In this season the challenge has been getting protective gear to keep safe. Everywhere these are not available even here in private hospitals because these things are very expensive. Personal protective equipment is not there or not readily available. Imagine a doctor attending to a mother who is giving birth and the mother has COVID-19, you should be really fully donned and protected but we don’t have them, they are expensive.* **Obstetrician, Private-Not-for Profit hospital**

**Inadequate handover and care to mothers (absence of healthcare workers)**

In relation to curfew and limited movements, there was a barrier of inadequate handover. Before COVID-19 and Lockdown, whenever a person was completing his/her schedule, they would first orient the incoming person what they have done and the next course of action. However, during this period, this was not possible as everything was done in a rush. Thus, there was no proper handover over as your time for going home approached, you would just run away without briefing the person who was going to take over.

*One day a mother delivered on the ward in the absence of a health worker you can imagine. Professionally you are not supposed to leave patients unattended to but because of COVID-19 we had no one to blame. That shows you there is a change in the quality of care.* **Midwife, Public hospital**

*Having no one to hand over was a challenge because staff had to leave early to beat the curfew even where handing over was not done, you needed to have continuity. For example, if you arrived and found a dead mother on the ward, what report would you give about this dead body and what happened. So when you arrive for your shift, I had to make sure I wake up every mother on the ward just to be sure there was no dead mother on the ward when I don’t know before taking up the shift [laughs].* **Midwife, Public hospital**

**Clients presenting without necessities, finances and safety materials**

There was also a barrier of financially constrained clients coming for service. Some of the participants revealed that there were some patients who presented to the hospital without any of the requirements. This made it hard because some of the facilities do not work on people without paying something especially the private facilities even those that are not for profit but require some user fees found it difficult to work on such patients.

*Some mothers would come without masks and they would complain that they don’t have money to buy masks, in the end they would want us to buy for them. Others could come to deliver when it’s their due time and they completely have nothing to use and all they would say is that they have no money. Remember they need to come with cotton, gloves, polythene sheets etc so as a staff you start struggling to find a way of helping such a mother because she tells you she has no money and indeed when you look at her she seems not to have it.* **Midwife, Public hospital**

**Limited time and interaction with health worker in order to meet curfew times**

In relation to curfew, there was a significant decline in the time between the patient and the health worker. This is because the health workers were always on a rush to beat curfew time on their way back home. This is because in as much as some of the health workers if not all had travel permits, some of the law enforcement officers were not as understanding to let them go without a hustle. Therefore, the limited time didn’t allow enough time with the patients and some of the issues were not discussed well.

*Patients come here and want to be seen in a short time, they tell you we don’t want to have security issues because we are not meeting the curfew time because they come from various locations far and near. Then still because of that the information you give to the mother cannot be adequate for our patients. So even now with the adjustment of curfew to 9pm, the time is still limited. Ehhh a patient you would give 30-40 minutes; you now give 15minutes* **Obstetrician, Private hospital**

*The barrier had to work on many patients in a short time such that you have enough time to walk home before the curfew. Sometimes it was risking our lives because we would leave late, move on foot and feared that any time thugs can attack me on the way* **Obstetrician, Private-Not-for Profit hospital**

**Challenges and Difficulties**

**Numerous Missed appointments**

Some of the challenges that the participant highlighted included missed appointments by the participants. This was due to the limited movement that came with the lockdown. Therefore, some of the patients got challenges back home as well as attending to traditional birth attendants. This in turn led to many emergencies during that time and they were bad.

*Appointments: patients would not keep appointments because they would not come as scheduled and they would give you all sorts of reasons as to why their appointments were not maintained. Therefore, that is a big* **Obstetrician, Private-Not-for Profit hospital**

**Limited protective gear and resources**

Most of the participants revealed having had limited protective gear. This is because it was un usual time as these protective gears were not as much emphasised as now. Therefore, this time it was difficult to work on a participant without protective gear as the following quotes suggest.

*"They gave us protective gear and basically that is it. There are times we were not having enough protective gear but they would make sure that there is at least something little to use."* **Midwife, Private-Not-for Profit hospital**

*You are on the ward, you have no mask or PPE, you are interacting with many mothers but they are telling you that COVID-19 is all over and also medical people are falling sick. You would sometimes fear for your life to go on the ward.* **Midwife, Public hospital**

*The PPE and sundries situation wasn’t that good because they were not enough. They would give us surgical masks like today and the following day they don’t because they are not enough, the gloves too weren’t enough to the extent that we reached a point of disinfecting a glove so that you can attend to more patients using the same pair of gloves. Sometimes sanitizers too weren’t enough.* **Midwife, Public hospital**

**Harassment from security operatives enforcing the curfew**

Majority of the participants reported having had an episode with the security officers on their way to work. They revealed that they were stopped and asked where they were going and were required to show up their identity cards for them to be released to go. While others got some beating from the law enforcement officers. Thus a very challenging situation for the health workers and almost jeopardised their work.

*"There was actually a time police officers stopped us especially the time when the curfew was at 7 Pm and it was coming to 8 Pm. They stopped us, we were ordered to come out of the car, and they told us to kneel down. So we all moved out and knelt down.* **Midwife, Private hospital**

*There is a day where I really worked, curfew caught up with me, and my colleague had not reached so I worked for 24 hours without a break. One time we were stopped and caned and you see even before you remove your ID to explain yourself you have received some kiboko [been beaten] by the time to tell him am a health worker coming from delivering a mother its too late you have already been beaten and that cannot be undone. We have been through all that.* **Midwife, Public hospital**

*"There are sometimes curfew would get me on the way before reaching home. These policemen could stop me on the way to ask why and where I am going. Then I would panic and quickly negotiate with them, give them 'chai' [bribe] because they can beat you up before you present your ID from the work place to show that you are a health worker.* **Midwife, Public hospital**

*By the way as health workers, we were also affected in terms of transport, as it was not easy to access the hospital as cars were grounded. Finding transport to the facility really affected our movements. In some cases, you had to explain yourself to the security organs as to where you were going and what you were going to do [laughs].* **Midwife, Public hospital**

**Community not following the guidelines**

Following community guidelines was also very hard because of the fact that some people in the community were not adhering to the Ministry of health guidelines. This meant that the health workers feared contracting COVID-19 from the patients. So this was also a challenge as the health workers were sceptical of their patients.

*The other difficulty is with the community because some people are concerned about COVID-19. You rarely find people on the streets putting on a mask or keeping the physical distance* **Midwife, Private hospital**

**Abrupt change in neonates or mother's health with absence of consultants**

The other challenge was changing in the health of women. This is because the expecting mothers were coming to the clinic late and in a bad condition. It would be very bad if the mother got complications and there is no consultant at that particular time to work on them. Thus having serious complication to a tune of even losing some mothers and their children. In as much as this was not a rampant case, it so happened that some of the mothers lost their lives.

*What I feared most is, you receive a mother she is very normal, she is labouring well, she delivers the baby and then after a few moments the baby changes… and the baby dies and then also secondly there are mothers after delivery you could have done everything right then a mother begins gashing blood like an open tap. Because we are few staff and less consultants, you can even take the whole week not settled because you will begin asking yourself if there is something you did wrong.* **Midwife, Private hospital**

*"contact between the patients and the health workers because the means of spread pf this disease, it is quite challenging, actually you are supposed to be very far from the patients yet some procedures you have to touch the patients even though you are putting on gloves and our patients also don’t want to put on masks, you put on them much pressure to put on masks but most of them, you find they have not come with them. So we get that challenge of working with a patient without a mask yet she needs our services. Some say they cannot put on masks because they can’t breathe well when the mask is on and some say that we are just wasting their time, the disease is not there* **Midwife, Private hospital**

**Mothers in labour not tested for COVID-19**

The other challenge was the fact that the mothers in labour are never tested for COVID-19 and this is distressing to the health workers. These mothers are the ones that cannot put on a mask in their condition. Thus one wonders whether these mothers are safe and if not safe, what would happen if they are infected. Therefore living in fear most of the time.

*But our challenge was it was difficult for us because mothers were coming pregnant and due for labour, they were not tested for COVID-19 but they were just left to come in because someone is in labour which was a big risk.* **Midwife, Private-Not-for Profit hospital**

*Unavailability of expensive COVID-19-91 tests to screen the pregnant mothers We are not testing those who come to deliver, we have never tested any single mother here. Remember we have limited testing of these patients so you cannot tell if they are COVID-19 positive or not. but remember they are coming from the community. It would have been good to test them when they come so that even if she delivers we know what to do. So we try to be vigilant even after giving birth to know our numbers like those mothers who were positive and see what to do. We are not testing because the test is expensive****.* Obstetrician, Private hospital**

**Absence of transport to facility - had to walk miles**

Absence of transport was also a major challenge. This is because some of the health workers come from very far and they found it challenging to access the health facilities. In as much as the facilities provided transport for the health workers, they were not able to take the health workers to their homes. So some found it difficult to move to the designated stages.

*Remember I was footing [walking] from the time COVID-19 lockdown till the easing of the public transport because some of us we never got transport. When I knew my shift was at 8pm, I would leave home and start walking at 3pm so I can get here on time also before it got dark.* **Midwife, Private hospital**

*Before COVID-19 the work was going on like it is today but we got a challenge of transport because where we stay far from our work place and transport fare itself was increased when they opened public transport. But before that we would walk, we would struggle to reach the health facilities and render some services. Pg 3 Ln 37-39 We had transport challenge, we could find that sometimes we could fail to come for work because the transport fare was high. So if you could try and come for work like today, then you could fail to come tomorrow because you don’t have enough money for transport. In the end we would not offer good services like we are supposed to because sometimes mothers could come and find just one health worker and they could wait for the whole day.* **Midwife, Public hospital**

*As I told you, transport is a big problem. During the lockdown, transport became very difficult and expensive. Ok even if you had the money, it was difficult to get a vehicle to get you here and yet you needed to come and work on the patients but no car to bring you and the same for our patients who missed their appointments.* **Midwife, Private hospital**

**Heavy work load due to limited number of health workers**

There was also a challenge of work overload due to the limited number of health workers at some facilities. This was because of the reduction in the number of staff coupled with transport challenge that made it impossible for the health workers to come. So for those who managed to come to the facility were overwhelmed by work as if one was meant to work on 10 patients, they would end up working on more than what they were supposed to work on.

*There was a heavy work load for those of us who managed to reach the facility because some staff would fail to come for work completely, so we would cover up for those who couldn’t make it here.* **Midwife, Private hospital**

**Short consultation times with no room for lengthy explanations and asking questions or inquiries**

There was also a challenge of limited space. Since COVID-19 required social distancing, there was a challenge because the rooms were designed to for a few people but were being used by many people. So there was a challenge as far as social distancing was concerned.

*when it comes to examination here, of course, we make sure we examine them to the best of our ability but within the shortest time possible. We don’t give them time to explain this and that, it’s strictly examination time. We examine very first and let them go.* **Obstetrician, Private hospital**

*_*

**Decline in quality of care due to staff shortage, stock out of essential medicines**

Decline in the quality of services offered to the mothers and their children was evident. This was because of the fact that there was limited time for patients and their doctors in a bid to reduce congestion and also beating time for curfew. Therefore, things would be rushed and most of the health workers thought that they were not getting enough with the patients and thus impacting on the quality of services.

*The quality of the service went down during lockdown. For instance, as an obstetrician, those mothers who have had surgeries, I feel am not giving them the best because of those drugs that are out of stock. Then also because of the number of staff was reduced, the few staff who are still working are overworked and cannot produce the best, so you find some things not yet done, some treatment not yet given, not because the people forgot but because they are too busy.* **Obstetrician, Private-not-for Profit hospital**

**Greatest Fears**

**contracting COVID-19 virus and infecting family members**

The greatest fears that almost all the participants acknowledged and reported was the fear of contracting COVID-19. This is because most of the participants did not know much about the disease and were fearful of the contracting it and taking it to their families. This was the greatest night mare of all health workers as the following excerpts show.

*"My greatest fear was contracting COVID-19 and spreading it to my little children. That is the most disturbing thing even up to now. I think about it most of the time even now. I have fear of everyone I work on.* **Midwife, Private-not-for Profit hospital**

*Contracting COVID-19 ohhh my greatest fear is catching the disease COVID-19 [laughs] but we have to serve, we have to serve because we are called to. I also keep wondering if the mother has COVID-19 can’t the baby also get infected ummmm because now you see how COVID-19 can be spread through droplets and the mother is in direct contact with the baby.* **Midwife, Private hospital**

*"Sometimes we don’t have PPE so for me if the blood flashes on me I have a hundred questions to myself. Am I really safe? Yet you are also scared to go for the tests. Actually, am told the COVID-19 test is also scaring and the disease leaves the scars in your lungs then you already know that you have a few days to leave.* **Midwife, Private-not-for Profit hospital**

*My greatest fear was contracting COVID-19 because you could not easily tell who was sick and who was not. They used to tell us some people can be infected with no clearly visible signs and symptoms. Then you also don’t have protective gear, you are dealing with a lot of patients, you imagine you are sharing facilities for example you would go to the lift and it would be full of people, on one is protected and you wonder. I feared everyone because I did not know their COVID-19 status and am sure others feared me too. There was no way you could avoid people here because once you enter a hospital you have gone public with people from all over and as a medical worker you cannot avoid it.* **Midwife, Public hospital**

*I had a fear that anytime I would also get infected with COVID-19 because much of the time I am with people coming from different places. So I would fear that anytime I would get affected as well with COVID-19. We also fear touching these mothers sometimes for fear of contracting COVID-19, much as the measures are there in place but you cannot rule out those doubts in us of how safe am I not to catch the virus?* **Midwife, Private-not-for Profit hospital**

*On my side, contracting COVID-19 while offering the service is my greatest fear because I go back home – contracted COVID-19 or not I don’t know but then I interact with my family and those at home. They can all contract the virus which is a problem and still on my side.* **Midwife, Private-not-for Profit hospital**

**Looming 2nd lockdown**

Some of the health workers also revealed that they were scared of the second lockdown. This would again shut down everything and people become stranded again as far as health seeking was concerned. So some of the health workers were also fearful of jobs and thought that when the lockdown is instituted again, most of their friends will be laid off at their work places as job security was uncertain. While others were looking at it in a business perspective that they were going to loose business.

*of course the other fear as the number of COVID-19 patients increases, we keep thinking are we going back for another lockdown, the hospital administration may cut the number of staff and we won’t be paid to care for our families. In the first two months of the lockdown they used to pay us half but it has now* **Obstetrician, Private-not-for Profit hospital**

*"Fear that time might come and patients are no longer coming here and you have nothing to do because it happened in the first two months of the lockdown and I expect it to happen again if there is another lockdown. If this happens, management will find no use for me so I will lose my job.* **Obstetrician, Private-not-for Profit hospital**

**Abrupt change in neonates or mother's health with absence of consultants & Loss of a mother or baby**

Like the challenges, the fear was the mothers’ health deteriorating when they are not under the care of the health workers. This was because the health workers thought that the patients would get worse from home and by the time they come to the health facility, they are beyond repair. So some of the health workers held this as a fear that they held during the time of the pandemic and the lockdown.

*What I feared most is, you receive a mother she is very normal, she is labouring well, she delivers the baby and then after a few moments the baby changes… and the baby dies and then also secondly there are mothers after delivery you could have done everything right then a mother begins gashing blood like an open tap. Because we are few staff and fewer consultants, you can even take the whole week not settled because you will begin asking yourself if there is something you did wrong.* **Midwife, Private-not-for Profit hospital**

*I don’t want to lose a mother and worse still a baby during this season. It’s one of the worst things you want to see happen. To lose a mother and a baby, I fear that greatly. That is on the side of the patients.* **Obstetrician, Private hospital**

**Job loss due to unpredicted long "forced leave"**

There was also a great fear of losing jobs completely by some health workers at some facilities. This is because of the fact that some of the health workers at some facilities were forced to take leave without pay. So some of these felt that due to COVID-19 and the lockdown that was imposed, they were going to lose their jobs which was yet another night mare for the majority of the health workers especially in private and not for profit facilities.

*I may be would fear to lose the job like we have seen for some of our colleagues got forced leave and living in suspense because you are not paid, you don’t know whether you have a job or you have been replaced which is a stressor. Also the salary would be reduced. Also you remember when the president mentioned that salaried employees need to contribute some money off their salary then I worried that my small money am earning is again going to be further deducted, so then I worried. It was not good.* **Obstetrician, Private-not-for Profit hospital**

*One of my greatest fear was losing my income in case I fell sick or in case I was taken to isolation. Because as a hospital, there is no compensation for anyone that is working on highly risky people or on anyone that has contracted the virus, As a specialist, I earn from consultation, each patient I see contributes to my income so if I go away for two weeks then am losing income.* **Obstetrician, Private-not-for Profit hospital**

**Falling in the hands of security organs while on-call**

Also getting at logger heads with security was also a great fear for almost all the health workers that were not staying at the health facilities. This is because they would be required to move back home and they were uncertain if they would beat the curfew time. This is because they would stop working a bit late and the law enforcement officers did not want to hear any form of explanations at some of the check points.

*Obstetricians who work in the night and you know our phones are always busy 24/7 since you have to be on-call, the fear to go through the night is too much. You find that they call you up at night, there is an emergency and you have to move from your home to the facility, there is virtually no one moving at that time of the night, you fall in the hands of the security organs who mistreat you, so it’s very very risky. At times you have to move to save a mother and her baby because you have nothing to do but with a lot of fear. So things have been affected.* **Obstetrician, Private hospital**

**Effects of the pandemic on work and quality of service**

**Involvement of various stakeholders and additional care in service provision**

Participants reported that some of the mothers came to the health facility without enough supplies during COVID-19 and lockdown. This is because they basically had no money. Our respondents recommended inclusion of all stakeholders in upholding the plight of the expecting others by increasing stock of supplies especially those used in delivery services. This also includes provision of all the protective gears ranging from the face masks to the overalls the mid wives put on.

*Mothers who come and they are in labour as you understand, we will provide from the ones meant to be for staff because we expect them to come with one because the hospital doesn’t cater for that but you know with labour [laughs] it catches you unaware and the mother comes saying I have forgotten the mask and there is nowhere she can buy it at that time so we give them especially the mothers. The attendant will have to go and look for one. But even for the attendant, there is no way you can enter through the gate without the mask. You see even staff are not allowed to enter without a mask. The first checkpoint at the gate requires you to have a mask. Then on the doorposts as you enter the ward you find askaris [security officers] who cannot allow you without a mask and without hand washing.* **Midwife, Private-not-for Profit hospital**

**Reduction in clientele attending the hospitals**

There was also an effect on the number of people attending to hospital services. This was attributed to limited transport and curfew. This is because before COVID-19 and the lockdown, people would move at any time to seek health services but during this time, people were not allowed to move freely and thus many resorted to walking to the nearby health facilities other than their usual health centres. This made some of the hospitals to have a few people attending their services.

*Well the COVID-19 situation has affected us seriously. The number of clients reduced since then which is not good for us in this period, and then the period changed because of the curfew time so people come in briefly compared to before.* **Obstetrician, Private hospital**

*The rate at which they receive family planning has also dropped because they don’t come back to the hospital because of COVID-19 so that kind of flow is disorganised. A mother would come for antenatal care and safe delivery then after delivery they would come for post-natal care and family planning at 6 days post-delivery and that has affected the flow of safe motherhood and how it should be. This can cause issues and possibly some may die in the community and we don’t know yet we could have helped.* **Midwife, Private-not-for Profit hospital**

*Resorting to TBA for delivery the patients reduced during the lock down. It was not because there were no patients actually they were more because people were at home but could not reach the hospital because of curfew, lockdown of transport or those who wanted to deliver and not hustle with getting a letter from the RDC would just go to a nearby traditional birth attendant and give birth from there other than struggling to get here.* **Midwife, Public hospital**

**Delayed service provision due to staffing shortage**

In some instances, there was some delay in service provision because of the delays in transport as health workers had to move from point to point of road blocks being stopped. They would spend a lot of time on the way yet there were emergencies waiting for them. This almost resulted in serious complications while some succumbed to this delay.

*I see that there is delayed service somewhere because the staff are few and the patients are sometimes very many. For example, you are 3 staff on the unit and you have like 8-10 deliveries, so there is a delayed service although it’s not always but it’s because of the few staff available.* **Midwife, Public hospital**

**Facility financial challenges**

There was also some financial challenges. This was not only on the side of the health workers and the patients but also to the hospitals as a whole. This was due to the reduction in the number of patients attending to these hospitals especially the private facilities as they depend on their clients for finances. This meant that those health workers who were able to work during the lockdown did not receive any bonuses or anything to motivate them other than their salaries.

*"The group that worked during COVID-19 for those three months their salaries remained the same, then the group that joined in later received half of their monthly salary. That is what happening to-date....is Yes, because like the hospital also has some little bit of financial problems due to COVID-19.* **Midwife, Private hospital**

**Changes in mode of service provision**

There was also significant change in the way the facilities started operating. This was in a way of observing standard operating procedures for preventing COVID-19. They started sanitising all the time, using gloves and masks even to so small procedures that they would not have otherwise used these protective gears. They also required the patients to hand wash. The health workers reported that in as much as hand washing and sanitising was meant to be their norm, it was not as frequent as it is today.

*We tell them to put on face masks, wash hands and sanitize but because we don’t have enough sanitizer to be sanitising themselves all the time we tell them to wash their hands and of course sanitisers would be the best because most people don’t want to wash hands all the time so sanitisers come in useful and then social distance. Actually as a health worker, we create that environment here and ask them to abide. Like we ensure there is enough space between their beds, we ensure we attend to one patient at a time yet before we could handle like 2 mothers in one go. To get medicines, we send one at a time, on the issue of food they were initially lining up, we now had to control them now we give them bed per bed one after the other.* **Midwife, Public hospital**

**Closure of key services at the facilities**

Closure of some services especially those that were relating to community out reaches and antenatal services in some facilities. This was because of the reduction in staff as well as lack of transport. This were services that were aimed at taking services closer to the people but were affected by COVID-19 and the lockdown respectively.

*I believe there were many mothers who suffered from their homes. Of course there were some departments that were closed like antenatal, family planning and immunisation and am sure there are those who needed those out-patient services and could not get and others only come here to get comprehensive package not only one activity.* **Midwife, Private-not-for Profit hospital**

**Recommendations**

**Resource facilitation to contact clients**

Some of the participants reported the need for more facilitation especially with things to do with communication services so that they can get in touch with their patients. This would make it possible for the health workers to know how the patients are and to organise an ambulance in real time. Therefore, improving their service delivery and also managing patients before any complication.

*"We should be facilitated with airtime to maybe call our clients and encourage them and also contact their peers because they are also there.* **Midwife, Public hospital**

**Door to door services during pandemics**

Most of the participants also recommended outreach services in future if such a pandemic happens. This was after a realisation that most patients missed their visits while others got complications from home. Participants believed that if services were taken closer to the people, it was going to make people’s lives better and also reduce tremendously the complications related to delayed service delivery.

*Maybe doing door-to-door services.* *This is done like twice a year and I think we should do it more often because of the pandemic most of the people may not make it to the hospital during pandemics and it would be better to find them home.* **Midwife, Private hospital**

*One of the key recommendations is to ensure that even in such times, the pregnant mothers can access services and health facilities at any time they can be given those toll-free numbers to call an ambulance to pick them up. If you were keen on the news, many mothers died and or lost their babies during the lockdown period because of transport. If may be the government can extend maternal services to all nearby health facilities whether Health centre II or III, that can help so the mother can access a service easily because during pandemics accessibility is the main issue.* **Midwife, Public hospital**

**Improving staff access to health facility/ providing transport**

Provision of staff with transport was recommended for all the health facilities. This was because what was provided this time was not comprehensive enough and a more robust one was recommended. This was also after a realisation that not all the health workers can be accommodated at the facilities yet all services have to continue.

*But I suggest that during pandemic times the hospital should be more prepared to reach its staff and bring them to work especially those who come from far. I know you cannot make all the health worker reside nearby- many have families to take care of..... Because if you receive a dying mother and you are trying to save her, you need the other colleagues –like consultants to be able to come to your rescue as soon as possible. The doctors and nurses are there and when we fail we refer.* **Midwife, Private-not-for Profit hospital**

**Adequate equipping of facilities**

Majority of the participants also recommended equipping all the health centres with enough supplies. This was because there were rampant shortages in protective equipment in some of the health facilities which was putting the lives of the health workers and their patients in double jeopardy.

*The health facilities should then be equipped with all the necessary supplies for obstetric emergency cases depending on the level of the health facility. Also we don’t normally get such big pandemics but this has taught us to be prepared and the hospitals need to be very prepared for such situations – have all the necessary equipment ready and on standby.* **Midwife, Private-not-for Profit hospital**

*We need proper PPE, for example, this period we need these gowns and gloves that are good although quite expensive. But we need to be seriously protected than we have been before. During delivery, we use a lot of gloves and we really need those.* **Midwife, Private-not-for Profit hospital**

*"The hospital in general needs to continue giving good services to the mothers and be well equipped with protective gear so we work without fear knowing that we are protected. They can also give us transport, which could help to pick up staff from their respective destinations to come and give services.* **Midwife, Private-not-for Profit hospital**

*One, we should be provided with PPEs like any other frontline workers and we also work in emergency situations. They should provide PPE to health workers who are not necessarily working on COVID-19 patients. What is really important is to have PPEs given to protect the health workers since they have decided to leave maternal health to continue operating and we work also with mothers who can be infected or may get infected. The minimum PPEs for the health workers working on mothers giving birth should be an apron, googles, long gyn. gloves, head gear and gumboots and that is the minimum. But where we feel the mother is a suspected case, we need the full protective gear and there should be no question about that* **Midwife, Public hospital**

**COVID-19 test added to routine care**

COVID-19 test was also recommended to be part of the normal routine at the health centres. This is because it was hard in a way that the patients were not screened for COVID-19. So there was nothing to do for the mothers who came in labour were not tested. Thus putting the lives of the health workers and fellow mothers.

*"I think for a mother coming to the labour ward, it should be a must that they are tested for COVID-19 just like we do for HIV and I think it should get in the routine because you never know whom you are dealing with. A mother may come when she is pregnant and you think that she is safe and yet she is not.* **Obstetrician, Private hospital**

**Provision of staff accommodation in proximity to health facility**

Most of the participants also recommended housing facilities.

*The government should build staff housing near the hospital such that in case something like this happened again we would not struggle. So that issues of transport, I did not find anyone on the ward to get a report because they are beating curfew is sorted. If we were in the quarters, we would have no reason to run off before time or not attend to our work or be worried about being beaten by the police men. Our service would remain the same because we are sleeping within the hospital.* **Midwife, Private-not-for Profit hospital**

*I would recommend hospitals to put up many hostels where health workers can stay during time of pandemic and it should be near the health facility. During the lockdown, we had very few rooms for accommodation but most staff where coming from outside and took a long time to arrive and had to leave earlier* **Midwife, Public hospital**

*I think the hospital needs to know that such things can happen and make sure that there is not only the budget but enough housing for health workers who can be housed within the hospital in case transport is completely locked down.* **Midwife, Public hospital**

**Gender based violence**

**Mistrust causing violence**

*"Some of my patients got problems with their spouses back home. There was some kind of domestic violence, and what was causing it was mistrust. Madam thought that the husband was taking ARVs and yet the husband was taking medication for Hepatitis. Therefore, because some of the drugs taken by HIV patients can be shared with people with hepatitis, it was hard for her to understand. He had Hepatitis even before the lockdown but I think the wife had not seen the medication the man was taking because the husband works from Sudan.* **Midwife, Private-not-for Profit hospital**
