# Supplementary material for: Lived experiences of frontline healthcare providers offering maternal and newborn services amidst the novel corona virus disease 19 pandemic in Uganda: A qualitative study
Source: PLoS One. 2021 Dec 10;16(12):e0259835. doi: 10.1371/journal.pone.0259835 (PMC8664167; doi:10.1371/journal.pone.0259835)
Supplement: Attachment — Submitted filename: Response to Reviewers PROM version 3.docx [file pone.0259835.s002.docx]

**AUTHORS’ RESPONSE TO REVIEWS**

**TITLE: Impact of Mode of Delivery on Pregnancy Outcomes in Premature Rupture of Membranes after 28 weeks of gestation in a low resource setting: A Prospective Cohort Study**

**Authors**

Herbert Kayiga (hkayiga@gmail.com)

Lester Felicia ([Felicia.Lester@ucsf.edu](mailto:Felicia.Lester@ucsf.edu))

Pauline Mary Amuge ([paulacallista@gmail.com](mailto:paulacallista@gmail.com))

Josaphat Byamugisha ([jbyamugisha@gmail.com](mailto:jbyamugisha@gmail.com))

Amy (Meg) Autry ([meg.autry@ucsf.edu](mailto:meg.autry@ucsf.edu))

**Version: 3**

**Date:** 20^th^December 2017

Authors’ response to reviews: See over

**20^th^ December 2017**

TO: **THE PLOS ONE EDITORIAL TEAM**

***Object:* MS:PONE-D-16-47299 Impact of Mode of Delivery on Pregnancy Outcomes in Women with Premature Rupture of Membranes After 28 Weeks of Gestation in a Low-resource Setting: A Prospective Cohort Study**

With great pleasure we are thankful for your consideration of our manuscript for publication in your reputable journal. In response to the editors’ comments sent to us on 20^th^ December 2017, we have revised the above manuscript accordingly.

**Journal requirements:**
1. Author Amy (Meg) Autry is listed as “Amy Meg Autry” in the online submission form; we are unsure which version of each name is correct. If the name is incorrect in the manuscript, please correct it. If the name is incorrect in the submission form, please let us know so we can correct the author’s profile for you. Please note that the use of middle initials/names should be consistent among the manuscript’s author list, the author list in the submission form, and the Author Contributions initials.
***Response:*** *Please help me edit the online submission to maintain ‘Amy (Meg) Autry’.*

2. In the References section, please list the first six author names (no more or fewer if the reference contains more than six authors) before including "et al." For further information, see our guidelines for references: <http://journals.plos.org/plosone/s/submission-guidelines#loc-references>. If you are using EndNote, please ensure that you use the most recent version of the PLOS output style, which can be found at <http://endnote.com/downloads/style/plos-public-library-science-all-journals>
***Response:*** *The references have been adjusted to meet the reference guidelines.*


3. Please combine Table 1a and 1b into a single table or rename them with different numbers (e.g., Table 1 and Table 2), and be sure to update the citations in the main text for all tables accordingly. Please feel free to refer to examples of tables here: <http://imgur.com/a/xurA2>

***Response:*** *Table 1a and 1b have been merged and named Table 1 in the updated manuscript.*

4. Your tables are not cited in ascending numerical order; currently Table 4 is cited before Table 2. Please cite tables in ascending numerical order in the body text of your manuscript.
***Response:*** *The tables are cited in an ascending order in the revised manuscript.*

5. Please list the label and legend for your figure in a section titled "Figure Captions" at the end of your manuscript. This section should immediately follow the References section and precede the tables. Please ensure that all labels of figures are in the format “Fig #” (e.g., “Fig 3”). Guidelines can be found at <http://journals.plos.org/plosone/s/figures#loc-captions>

***Response:*** *The title and legend of figure 1 have been added in the revised manuscript.*

6. Your figure is not the correct file extension type. Please ensure that all of your figures are in either TIF or EPS format, are between 300 and 600 DPI, and have a width of at least 6.68 cm. Figure guidelines can be found at <http://journals.plos.org/plosone/s/figures>. If you need assistance with your figures, please contact [figures@plos.org](mailto:figures@plos.org).

***Response:*** *The cited figure is now in the form of TIF.*
