# Supplementary material for: Lived experiences of frontline healthcare providers offering maternal and newborn services amidst the novel corona virus disease 19 pandemic in Uganda: A qualitative study
Source: PLoS One. 2021 Dec 10;16(12):e0259835. doi: 10.1371/journal.pone.0259835 (PMC8664167; doi:10.1371/journal.pone.0259835)
Supplement: Attachment — Submitted filename: Response to reviewers COVID 4.docx [file pone.0259835.s003.docx]

**AUTHORS’ RESPONSE TO REVIEWS**

**TITLE: Lived Experiences of Frontline Healthcare Providers offering Maternal and Newborn Services amidst the Novel Corona virus Disease 19 Pandemic in Uganda: A Qualitative study**

**Authors**

Herbert Kayiga (hkayiga@gmail.com)

Diane Achanda Genevive ([achandadiane@gmail.com](mailto:achandadiane@gmail.com))

Pauline Mary Amuge ([paulacallista@gmail.com](mailto:paulacallista@gmail.com))

Andrew Sentoogo Ssemata ([andrewssemata@yahoo.co.uk](mailto:andrewssemata@yahoo.co.uk))

Racheal Samantha Nanzira (nanzirasamanthar@gmail.com)

Annettee Nakimuli ([Annettee.nakimuli@gmail.com](mailto:Annettee.nakimuli@gmail.com))

**Version: 4**

**Date:** 9^th^ August 2021

Authors’ response to reviews: See over

1^st^ **September 2021**

TO: **THE PLOS ONE EDITORIAL TEAM**

***Object:* PONE-D-21-03659 Lived Experiences of Frontline Healthcare Providers offering Maternal and Newborn Services amidst the Corona virus Disease 19 Pandemic in Uganda: A Qualitative study**

With great pleasure we are thankful for your consideration of our manuscript for publication in your reputable journal. In response to the editors’ comments sent to us on 5^th^ August 2021, we have revised the above manuscript accordingly.

**Editor’s comments**

Journal Requirements:

Please review your reference list to ensure that it is complete and correct. If you have cited papers that have been retracted, please include the rationale for doing so in the manuscript text, or remove these references and replace them with relevant current references. Any changes to the reference list should be mentioned in the rebuttal letter that accompanies your revised manuscript. If you need to cite a retracted article, indicate the article’s retracted status in the References list and also include a citation and full reference for the retraction notice.

Response: *Thanks for the comment. The reference list has been revised accordingly.*

Additional Editor Comments (if provided):

I suggest paying careful attention to all three reviewers' comments, but would specifically like to highlight:
1) The need to use consistency in language (e.g., COVID, COVID-19, Corona Virus). After defining the Coronavirus disease (in the first paragraph of the introduction), please select one term and use consistently in the abstract and text.
Response: *Thanks for the recommendation; COVID-19 is now the consistent term used in the revised version of the manuscript.*
2) The first two references are about the United States. Please ensure you are using appropriate global resources (ideally from the WHO) when describing the global pandemic.
Response: *The first two references (Garg, S., et al., Hospitalization rates and characteristics of patients hospitalized with laboratory-confirmed coronavirus disease 2019—COVID-NET, 14 States, March 1–30, 2020. Morbidity and mortality weekly report, 2020. 69(15): p. 458.*

*2.	Team, C.C.-R., et al., Geographic differences in COVID-19 cases, deaths, and incidence—United States, February 12–April 7, 2020. Morbidity and Mortality Weekly Report, 2020. 69(15): p. 465-471) have been replaced with new references as published by the World Health Organization (Sohrabi, C., et al., World Health Organization declares global emergency: A review of the 2019 novel coronavirus (COVID-19). International journal of surgery, 2020.* ***76****: p. 71-76.*

*2.	Purcell, L.N. and A.G. Charles, An Invited Commentary on “World Health Organization declares global emergency: A review of the 2019 novel Coronavirus (COVID-19)": Emergency or new reality? International journal of surgery (London, England), 2020.* ***76****: p. 111).*

It is not a journal requirement to include data (especially if potentially identifiable). I agree with Reviewer #2 that there are a number of factors that compromise the confidentiality of the sample (e.g., the raw data file, listing the hospital/health facility names and locations, specificity about participant characteristics per site). I would recommend removing Table 1 and summarizing characteristics of the type of facilities included (but not needing to name them).

Response: *Thanks so much for the comment. Table 1 from the original draft was removed and revised as recommended in the Rebuttal letter of 7^th^ June 2021. The current table 2 doesn’t reveal the identity of the participants.*

I would summarize Table 2 to list characteristics overall (e.g., number of males, females; range and mean for age, etc.) based on hospital type as recommended by Reviewer #4. Consider removing the individual names of hospitals and thanking the administrators more generally. Finally, it is not necessary to submit ethical approval forms, interview questions, and the raw data. I would consider removing these.

Response: *Thanks for the recommendation. The ethical approval forms, interview questions and the raw data will be removed.*

4) Please note the suggestions to the rigor section described by Reviewer #2.
Response: *The suggestions have been noted.*
5) Table 3 is an excellent addition to the manuscript.
Response: *Thanks for the observation.*
We look forward to receiving your revised submission.


**Reviewers' comments**:

Reviewer's Responses to Questions

**Comments to the Author**:
1. If the authors have adequately addressed your comments raised in a previous round of review and you feel that this manuscript is now acceptable for publication, you may indicate that here to bypass the “Comments to the Author” section, enter your conflict of interest statement in the “Confidential to Editor” section, and submit your "Accept" recommendation.

Reviewer #2: (No Response)

Reviewer #3: (No Response)

Reviewer #4: (No Response)

2. Is the manuscript technically sound, and do the data support the conclusions?

The manuscript must describe a technically sound piece of scientific research with data that supports the conclusions. Experiments must have been conducted rigorously, with appropriate controls, replication, and sample sizes. The conclusions must be drawn appropriately based on the data presented.

Reviewer #2: Partly

Reviewer #3: Yes

Reviewer #4: Yes

3. Has the statistical analysis been performed appropriately and rigorously?

Reviewer #2: N/A

Reviewer #3: N/A

Reviewer #4: N/A

4. Have the authors made all data underlying the findings in their manuscript fully available?

The [PLOS Data policy](http://www.plosone.org/static/policies.action#sharing) requires authors to make all data underlying the findings described in their manuscript fully available without restriction, with rare exception (please refer to the Data Availability Statement in the manuscript PDF file). The data should be provided as part of the manuscript or its supporting information, or deposited to a public repository. For example, in addition to summary statistics, the data points behind means, medians and variance measures should be available. If there are restrictions on publicly sharing data—e.g. participant privacy or use of data from a third party—those must be specified.

Reviewer #2: Yes

Reviewer #3: Yes

Reviewer #4: No

5. Is the manuscript presented in an intelligible fashion and written in standard English?

PLOS ONE does not copyedit accepted manuscripts, so the language in submitted articles must be clear, correct, and unambiguous. Any typographical or grammatical errors should be corrected at revision, so please note any specific errors here.

Reviewer #2: Yes

Reviewer #3: Yes

Reviewer #4: Yes

6. Review Comments to the Author:

| **Reviewer Comment** | **Response to Comment** | **Page No. and Line** |
| --- | --- | --- |
| Reviewer 2#  Many improvements evident throughout, most notably the addition of Table 3 2. Key words: suggest including "lived experiences"  3. Publication Ethics Table 1: by mentioning the names and identifying information of each study hospital continues to reveal who the study participants were and compromises their confidentiality. Please delete hospital names and identifying location information (highway or hill name, founding details, division name; does it matter in this case whether it was Anglican or Catholic?). Keep in minimal information that helps the reader understand what type of hospital with what type of capabilities and catchment was studied.  4. Publication Ethics: Same is true of the supporting information linked to the manuscript. Other than the participant interview guide, the documents contain hospital identifying information and therefore inappropriate to share with the journal’s readership. This includes: a. Clearance forms b. Protocol c. Consent form health workers d. Raw data set – every quote in the data set shows which hospital it came from; if you have to share, then all data should be de-identified, like you did in quotes used in the results section. This is a lot of work – I would rather you did not share due to data confidentiality issues. e. The other forms are not needed to be made public either and should be omitted from the supporting information.  5. The TASO IRB approval letter and the study UNCST Ref Number do not contain identifying information but they are not needed by the readership either and should also be omitted as unnecessary.  6. “All participants were sanitized” A better way to say this for people is that they practiced a disinfection protocol prior to the interview and hopefully their interviewers did that as well.  7. “Team 3 was composed of two independent 161 researchers whose task was to read and review the content (interview transcripts, and field 162 notes word-for-word, line-for-line) several times for quality checks and triangulation.” Do you mean to review transcripts and notes compared to the recordings for transcription accuracy? Team 2 was already doing that. I suggest you rephrase the description of Team 3 to say and leave it at that because you explain those further down. What you say about triangulation fits better under rigor – move it there and say how team 3 was doing triangulation of the analysis findings comparing data from one hospital with another hospital (because I haven’t seen any other method used for triangulation).  8. “The research materials were kept under restricted access by only authorized staff for 182 patient confidentiality and privacy” There were no patients in the study. Please clarify that it was about the confidentiality of the participants, now violated by Table 1 and supporting documents.  9. “Participants were reimbursed for participating in the study in form of transport refunds and 190 refreshments.” Perhaps clarify that the refreshments were to take home? They were all in masks and PPE.  10. “The interviews were transcribed verbatim immediately. Transcription accuracy was ensured 198 at the end of the interviews by the Principal investigator and one Administrator. Field notes 199 and the transcription were compared for congruency. Data collection and analysis were 200 conducted concurrently until data saturation was achieved. This was done so that insights 201 from the data analysis could be used to make the required adjustments in the interview guide 202 and evaluate the credibility of the emerging themes in the subsequent interviews.” This was already stated under quality control. Delete the repetitions and merge the rest with that section.  11. “Data was 203 coded and analyzed manually using a framework matrix developed using an Excel workbook.” This statement implies that you developed the codes deductively and put them into a pre-designed framework. It is in contrast with the detailed description right below on how you developed inductive codes according to phenomenology. Did you perhaps built the framework matrix in excel after that detailed and careful process? State that and move that sentence after the description of the code development, to line 215.  12. “We ensured long term 220 involvement of the research team with the healthcare providers.” What rigor criterion does this fulfill? I suggest delete unless you use it to say that you are confident that you established good rapport with the participants.  The authors did not follow my previous feedback on how to painlessly summarize rigor. I therefore provide feedback below on how to improve the accuracy and readability of the rigor section:  13. “Data dependability was 221 ensured by having team 3 that was devoted to continuous reading through of the transcripts to 222 ensure ongoing comparison of the key information generated during the data collection and 223 analysis processes.” This is about ensuring that the findings of the analysis were aligned with the data collected in the transcripts and is what you do for the credibility criterion. Please edit.  14. “Dependability was observed by the stringent coding procedure and inter 224 coder corroboration.” Here please add that you made sure to document what each code meant in detail as illustrated in Table 3, so that another researcher could replicate this coding under a similar context (dependability criterion).  15. “Data transferability was observed by ensuring that participants’225 statements were captured with barely any modifications made yet ensuring a rich, thick 226 description of the study process by the research team.” The first part of this sentence is about confirmability by capturing the statements of the participants without any modifications and use of quotes as you do in the results section. The second part of the sentence about the thick description of the study context is what you provide in the intro and tables 1 and 2 so the findings could be transferable by the reader to another context if it were similar to the one of the hospitals in Kampala (transferability criterion). Please edit so it is clear to the reader.  16. “Thorough checks of procedures and 227 results were emphasized to improve the dependability and transferability of the data [19].” This is good but if you clarify the sentences above it you don’t need to repeat.  17. “Confirmability was observed by comparing the results to other evidence and field notes by an 229 external investigator [34] who read and compared the study results with the field notes and 230 memos.” Comparing the results with other evidence is triangulation. Either say what other evidence you were comparing the results with or delete. The rest about comparing the results with field notes and memos is confirmability - merge with the other part on confirmability. But this is the first time you mention memos. Say under data analysis how you were also writing memos and for what purpose, or delete memos from the sentence.  18. “We ensured that the coordinators of the interviews or discussions didn’t participate in 231 the analysis but critiqued the results from the analysis and ensured that these results 232 conformed to their expectations from the discussions.” It is not a criterion of rigor that field researchers stay away from the analysis. What you did is “member checking”, i.e. another way of validating the findings. Please state.  19. “Field notes and transcripts, codes and 233 their interpretations were made by separate teams of investigators.” Again this is not a criterion of rigor. You can delete. Or merge under data analysis.  20. “More than 90% of the healthcare 239 providers…” You should not use percentages when the denominator is so small (n=25). Simply say the great majority of participants…  21. Publication Ethics Table 2: too much information that compromises the confidentiality of the participants and doesn’t improve the dependability and transferability of the results. Please look at my suggestions in the previous rounds on how to improve Table 2. You can collectively report average age and say that all had bachelors degrees and up. Why is marital status relevant here? Delete.  22. “The participants 242 generally had a number of similar experiences in regards to maternal and newborn health 243 service delivery irrespective of the nature of health facility they worked (Table 3).” Here is where you can say that your data comparisons during rigor analysis showed a number of similar experiences irrespective of the health facility.  23. Table 3 is very good and helps a lot. “Much stigma was associated with contracting COVID-19. This meant no working for more than 2 weeks for the infected healthcare providers.” This statement needs clarification. How could infected providers work for 2 weeks?  24. “A special773 vote of thanks goes to the Administrators of the eight health facilities namely; Kawempe774 National Referral hospital, Kawaala Health Centre III, China Uganda Friendship Hospital,775 Naguru (Naguru Hospital), St. Francis Hospital Nsambya, Lubaga Hospital, Mengo Hospital, 776 Kampala Hospital, and Case Hospital for all the support they gave us during the study period.” Here again you are compromising the confidentiality of your participants without improving dependability or transferability of the results. You can instead anonymously thank all the participating hospitals.  25. References 1 and 2 continue to be inappropriate. You can’t use studies from the US to support a statement of WHO. You need a WHO reference for that.  26. References 6, 7, 8, 10, 11, 14, 15, 37, 39, 40, 44, 49, 51 need a web link and date accessed 27. Reference 8 and 13 are the same reference. Eliminate one of the 2 and provide weblink and date accessed.  28. References 21-29 are not needed when you delete the hospital names.  29. References 38, 41, 50 missing volume issue page info. 30. A recommendation on how to best address the reviewers’ comments without missing any, is to create a two-column table where on the one side you list each comment and on the right side you insert your response and direct quote from the manuscript. | Thanks for the kind observation.  “Lived experiences” has been added to the key words  The hospital names and identifying location information has been deleted from the revised manuscript as recommended.  Documents containing hospital identifying information, clearance forms, protocol, consent forms and raw data sets will not be shared as recommended.  The UNCST, TASO IRB forms will also be retrieved as recommended by the reviewer.  The manuscript has been edited accordingly. It now appears as “*Disinfection protocols were observed prior to the interviews*.  The write up has been edited accordingly as advised by the reviewer. It now appears as “*Team 3 was composed of two independent researchers whose task was checking rigor according to the Lincoln – Guba criteria”.*  The manuscript has been edited accordingly as advised by the reviewer. It now appears as “*Triangulation was checked by team 3 that was devoted to continuous reading through of the transcripts to ensure ongoing comparison of the key information generated from one hospital to another during the data collection and analysis processes”*.  Correction has been made in the revised manuscript. The hospital details and supporting information has been edited as recommended. This now appears in the revised manuscript as “*The research materials were kept under restricted access by only authorized staff for participant confidentiality and privacy”*  Thanks for the keen observation. Since the participants were paid for both, to avoid the confusion, in the write up, refreshment has been deleted. It now appears as “*Participants were reimbursed for participating in the study in form of transport refunds*”.  The portion has been deleted as recommended by the reviewer.  Thanks for the recommendation. The manuscript has been edited accordingly. It now appears as “*Data was coded and analyzed manually using a framework matrix developed using an Excel workbook built after a detailed and careful process of the merging codes*’.  The sentence has been deleted as recommended by the reviewer.  Thanks again for the kind comment. The manuscript has been edited accordingly. It now appears as “*Triangulation was checked by team 3 that was devoted to continuous reading through of the transcripts to ensure ongoing comparison of the key information generated from one hospital to another during the data collection and analysis processes*”.  Thanks for the advice; this manuscript has been revised to “*We made sure to document what each code meant in detail as illustrated in Table 3*”.  As advised by the reviewer, transferability has been changed to confirmability as the sentence captures this aspect as opposed to transferability.  The revised manuscript now appears as “*Data confirmability was observed by ensuring that participants’ statements were captured with barely any modifications made. Data transferability was ensured by the research team so that a rich, thick description of the study process was documented to enable replicability in a similar context elsewhere*.  Revision has been made in the sentences above as advised by the reviewer. To avoid repetition, the sentence has been deleted from the revised manuscript.  The aspect about confirmability has been deleted as advised  This sentence has been removed from the section on “Rigor” and shifted to ‘Quality control” since it’s not relevant under rigor.  It now appears as *“We ensured that the coordinators of the interviews or discussions didn’t participate in the analysis but critiqued the results from the analysis and ensured that these results conformed to their expectations from the discussions. This was done to validate the study findings and also ensure quality in the study*”.  The sentence deleted as advised.  Thanks for the caution; the sentence has been revised accordingly to “*The great majority of healthcare providers had more than ten years’ experience offering maternal and newborn health services*”.  Table 2 has been improved as recommended by the reviewer. Marital statuses, level of education and age have been deleted accordingly. Age has been reported based on the average age and level of education as advised.  The sentence has been improved as advised by the reviewer. It now appears as “*Data comparisons during rigor analysis showed a number of similar experiences in maternal and newborn health service delivery irrespective of the health facility (Table 3)”.*  Clarity has been added to the write up in the revised manuscript. It now appears as “*Stigma was associated with contracting COVID-19. Infected healthcare providers were side-lined for two or more weeks. This meant no earning during the period of self-isolation or quarantine”.*  Thanks for the keen observation. The manuscript has been edited accordingly. This aspect now appears as “*A special vote of thanks goes to the Administrators of the health facilities that participated in the study*”.  Thanks for the advice. WHO references have been used for reference 1 and 2.  *1. Sohrabi, C., et al., World Health Organization declares global emergency: A review of the 2019 novel coronavirus (COVID-19). International journal of surgery, 2020. 76: p. 71-76.*  *2. Purcell, L.N. and A.G. Charles, An Invited Commentary on “World Health Organization declares global emergency: A review of the 2019 novel Coronavirus (COVID-19)": Emergency or new reality? International journal of surgery (London, England), 2020. 76: p. 111.*  Thanks so much for the advice. A web link and date accessed have been added in the revised manuscript  References 21-29 have been deleted from the revised manuscript  The volume and page info has been added as advised.  A 2-column table has been created as recommended by the reviewer to address the raised comments and their responses. | Page 3, Line 59  Table 1: Page 8, line 169,170  Page 15, Line 182-183  Page 16, Line 202-203  Page 18, Line 263-266  Page 17, Line 229-231  Page 17, Line 238  Page 17, Line 218-231  Page 18, Line 255-257  Page 18, Line 263-267  Page 18-19, Line 267-268  Page 19, Line 268-271  Page 17, Line 224-227  Page 19, Line 278-279  Page 19, Line 280  Page 19-20, Line 281-283  Page 20, Line 288-290  Page 41, Line 754-755  Page 42, Line 789-795  Page 42-45, Line 789-963  Page 44-45 |

Reviewer #3: 

| **Reviewer Comment** | **Response to Comment** | **Page No. and Line** |
| --- | --- | --- |
| Reviewer #3  Thank you for submitting this revised paper. You have obviously undertaken extensive revisions. My comments are only minor. These include: a) Introduction: i) Good to benchmark the extensive lockdown and other measures in Uganda at the start of the pandemic to those seen in other Africa countries (Ogunleye OO et al. Response to the Novel Corona Virus Pandemic Across Africa: Successes, Challenges, and Implications for the Future. Frontiers in pharmacology. 2020;11:1205) helping to reduce mortality - certainly when compared to e.g. a number of Western European countries  ii) Good to include more up-to-date figures for COVID-19 than late January. In addition % in WHO Africa vs. rest of the world (this builds on i)  iii) Lines 84 - 85 - I assume you mean 'Uganda' by 'U'. In addition - I do believe Uganda was more prepared than a number of other countries including e.g. US    iv) Line 91 - A similar situation on reduced routine vaccinations across Africa - please see Abbas K et al. Routine childhood immunization during the COVID-19 pandemic in Africa: a benefit-risk analysis of health benefits versus excess risk of SARS-CoV-2 infection. The Lancet Global health. 2020;8(10):e1264-e72  v) Line 104 - avoid unscientific terms such as 'grossly' throughout the paper - better to say 'appreciably' than 'grossly'  b) Discussion - I would concentrate on the key areas as well as say what the authorities in Uganda should now do as a result of your findings for this and future pandemic. This does not come through clearly enough. This does not mean adding to the Discussion - merely making it more focused. The same applies to the Conclusion. This would enhance the utility of the paper in Uganda, across Africa and across LMICs | Thanks for your kind remarks on the progress of the manuscript.  The manuscript has been revised carefully to highlight the measures that were undertaken in other low and middle income settings at the start of the pandemic. This now appears in the revised manuscript as “*With lessons from other low and middle income settings, like Vietnam where lockdowns, extensive contact tracing and social distance had resulted in barely any mortality attributed to COVID-19[7, 8], Uganda instigated a nationwide lockdown to contain the COVID-19 pandemic.*  *With no clear cure to COVID-19 [9, 10], like other African countries like South Africa, Malawi, South Sudan, Kenya, Ghana, Nigeria, and Rwanda[11], Uganda took a number of measures to contain the COVID-19 pandemic.*  Thanks for the caution. Up-to-date figures have been used in the revised manuscript. It now appears in the revised manuscript as “*As of 10th August 2021, globally there were 202.1 million confirmed cases of COVID-19 and 4.29 million deaths [3]. In Africa as of 10th August 2021, there were 5.14 million confirmed cases and 122,025 deaths from COVID-19, which is lower than the 78.6 million confirmed cases and 2.03 million deaths in Americans [3] and 61.2 million confirmed cases in Europe with 1.23 million deaths from COVID-19[3].*  *Uganda reported her first COVID-19 case on the 21st March 2020[4]. Since then the number of confirmed cases had reached 95,723 as of 06th August 2021 with 2,783 deaths reported by the Uganda Ministry of Health [5].*  ‘U’ is now written in full in the revised manuscript. The manuscript has been edited accordingly to justify the implementation of the lockdown in Uganda. It now appears “*With lessons from other low and middle income settings, like Vietnam where lockdowns, extensive contact tracing and social distance had resulted in barely any mortality attributed to COVID-19[7, 8], and more case fatality rates from COVID-19 in the United States [9] and Europe [10]where preventative measures were not fully implemented, Uganda instigated a nationwide lockdown to contain the COVID-19 pandemic.*  The manuscript has been edited accordingly. The write up now appears as “*COVID-19 has exerted enormous pressure on National Health Service programs in many African countries like Expanded Program on Immunization [17] as result of closure of some of the vaccination clinics with some of the healthcare providers put in quarantine when suspected or confirmed with COVID-19 or shifted to manage COVID-19 patients[13].*  *Despite evidence of routine childhood immunization benefit over COVID-19 associated risks with the vaccination clinics[18], the Ministry of Health of Uganda has already reported a decline in the current immunization coverage during the COVID-19 pandemic [19]. Similar trends in immunization coverage have also been reported in South Sudan, Zimbabwe, South Africa and Nigeria [13].*  Thanks so much for the advice. ‘Grossly’ has been changed to ‘appreciably’ in the edited version of the manuscript.  The discussion and conclusion write up has been edited as advised by the reviewer. | Page 5, Line 89-93  Page 4, Line 65-74  Page 4, Line 74,84-96  Page 6, Line 125-133  Page 7, Line 147  Page 36-40 |

**Reviewer #4:**

| **Reviewer Comment** | **Response to Comment** | **Page No. and Line** |
| --- | --- | --- |
| It is a pleasure to review the study entitled : “Lived Experiences of Frontline Healthcare Providers offering Maternal and Newborn Services amidst the Novel Corona virus Disease 19 Pandemic in Uganda: A Qualitative study”. This paper’s strength is in the richness of the data and the in-depth descriptions of the challenges faced by maternal and newborn healthcare providers in Uganda during the pandemic, and how that influenced care provision. The study is also a platform to raise healthcare providers’ voices about the horrible experiences and negative treatment that they received, and to share their opinions of recommendations to continue care provision during the pandemic and beyond. It is also difficult not to appreciate the rigorous research methodology that was applied. Despite the witnessed improvements in the structure of the manuscript after the first revision, there remained some issue that can be addressed before publication.  My two main comments are: - Although I do believe that the rich and expressive quotes are a strength of the manuscript, they do tend to make the results’ section a lot longer than it can be. Some of them are particularly long and repetitive of the text summarizing the results and therefore can be either shortened or deleted altogether (for example the one in line 300 – 304 can be deleted). Perhaps keeping one quote per theme is sufficient, and the reader can always refer to the “raw data” supporting information for more.  - The manuscript describes the lived experiences during the COVID-19 pandemic. Yet, as we all have witnessed, the pandemic has been ongoing for almost 16 months, and with varying levels of restrictions over time. In the manuscript, the time frame of the described “lived experience” is not clear: was it the early phase of the pandemic (first lockdown), or does it stretch to include the period of data collection? The authors can be more specific about the recall period. This can be very relevant especially considering the second lockdown that Uganda is recently going through, to see whether any of the lessons learned from the first lockdown have helped in managing the second response.  Other minor comments are noted below, divided by section: Supporting information: - File called “Raw data” : suggestion to change the name of the file to : ”Detailed summary of the data by theme with quotes” since it is not possible to share the raw data (i.e. complete transcripts of interviews) due to issues of privacy and anonymity. Naming the file “Raw data” gives the false impression that the dull transcripts are actually published. - File called “Participant interview guide” : it seems that this file contains questions addressed to women who have sought care and not to healthcare providers, and the questions do not match those mentioned in the response to the reviewers. Suggestion to please revise and align. Abstract - Suggest to rephrase this sentence: “With the travel restrictions, social distancing associated with the containment of the virus, the maternal and newborn healthcare service in Uganda could be inaccessible, unaffordable, and unavailable to both the healthcare providers and many pregnant or laboring women.” It seems like the care is unaffordable and unavailable to healthcare providers - Is this intentional? – consider using the space in the abstract to focus mainly on the barriers faced by healthcare providers  - This is a qualitative study and usually do not use terms such as “primary outcome” (which has more of a quantitative connotation). It is already clear in the objectives what the “outcome” of the study is. Suggestion to rephrase as: “the interview guide primarily explored xxxx” - The first sentence in the conclusion is probably correct but it is not a direct observation of this research. The conclusion can focus more on healthcare providers’ wellbeing and ability to provide care, and the need to respect and support them rather than about the service delivery  Background: - Line 84-85: The COVID-19 pandemic took U by surprise[8, 10]. o Although I agree, it did take “me” and everyone by surprise, but I think the authors mean “took Uganda” o Suggest to move this sentence to the beginning of the paragraph  - Line 93-96: “Interruption in access to quality maternal and newborn health services with the travel restrictions in place to curb the COVID-19, could put over 10,000 lives of both women and their babies in danger every single day of the COVID-19 pandemic.” o Please provide a reference to this estimate  o This paragraph could use a bit more information about the MNH situation in Uganda before the pandemic: e.g. maternal mortality rate, skilled birth attendance, facility birth coverage, ANC coverage etc. how did these aspects evolve over time? And why is COVID-19 a particular threat to them, especially if it’s affecting healthcare providers.  o The background is also missing information about the structure of the health system in Uganda before the pandemic – where do women usually seek care (hospitals, healthcare centres?) how is care covered? Public vs. private sector role in the health system? And how are they similar/ different to each other? etc.    - Line 98: there is a “15” misplaced after December 2020. Also not clear what this sentence adds: “if it means that healthcare workers were infected with COVID-19”? Please clarify , with more details about the number of healthcare workers if that is possible.  Methods: - Line 121: is this a public health facility? Not a hospital?  - Table 1: o Suggest to present similar and complete information on all the hospitals ; e.g. why is number of deliveries per year available for Kawaala health centre and not others? If possible recommend to add for all o Suggestion to divide the “description” column into more structured columns, for example: level of care (primary, secondary, tertiary) ; some proxy of size of the health facility (e.g. number of maternity beds or number of deliveries in the past year – depending on which info is readily available); number of maternal and newborn healthcare providers (total or estimate); operating hours; free vs. paid services o An important characteristic to mention about the hospitals is whether or not they treated any pregnant women / women in labour who were suspected/confirmed with COVID-19  - Explain a bit more about the selection of the hospitals (purposive sampling of the biggest hospitals in Kampala, from three sectors public, private, private not-for-profit) - Line 121 – 123: “These eight facilities were the biggest service providers for public and private maternal and newborn health care in Kampala.” – why past tense here “were”? Suggest to change to present  - Line 139: “All participants were sanitized”. Suggest to rephrase to : participants sanitized their hands or strict hand washing and sanitization were required from all participants…  - It is important to mention where the interviews took place in the methods section: was it at the health facilities where participants worked? Or at the researchers office?  - Line 155: “potential clients”. Suggest to rephrase to potential participants  - Line 168: do the authors mean: no new emerging themes?  - Quality control: what happened with the data from the pilot interviews? Was it included in the analysis? Please be clear about that and if yes why? If no why not?  - Line 180: data were backed-up? Where and how?  Results: - Title of the heading: please remove “baseline”  - Table 2 is not completely showing on the page (please format and resize) - Please align the use of “obstetrician/gynecologist” vs. “medical doctor” when describing the cadres in the methods, results and table 2 - Suggestion for table 2: to switch the rows and the columns (the three types of facilities become columns, so that the categories of each variable are not repeated every time) Private Public Private not for profit Sex Male 2 2 2 female 4 8 7 Age 20-29 0 1 1 30-39 4 5 4 >40 2 4 4  - It is useful to know how many interviews were done per facility – perhaps could be added to table 1?  - Table 3 – it is not clear for the reader why the page number is added to the table. Also keeping in mind that this might change when the paper is published, I suggest removing this column. Or it can be used to indicate a reference to the “raw data” supplementary material if necessary  - The authors indicate that HCP experienced a number of similar themes across the facilities, but did they note any discrepancies or similarities within the health facilities? E.g. differences between cadres who work at the same hospital? (just our of curiosity about dynamics between different cadres)  - The perception that patient numbers increased is interesting- despite the fact that we could have assumed the opposite to happen (blocked roads/fear of healthcare seeking in facilities).  - Comment/suggestion: try to avoid “quantitative” terms in the results (e.g. change “a significant number” on line 254 to something like “many/most” etc. - Line 258: exclamation mark after “gloves”. Suggest to remove to the keep the results description as objective as possible. - Suggestion to always refer to it as “COVID-19”. Sometimes COVID alone is used (it is ok if it’s in a quote, but not the main text). Line 451: suspected to have COVID-19 - Suggestion to spell out CME in-text (first occurrence line 529)  Discussion - Line 650-651: specify which services exactly  - Paragraph lines 685-697: recommendation about telemedicine should be considered with caution as it can lead to inequality in accessibility (poverty, illiteracy among women) and its impact on the quality of maternity care is not yet well understood.  Suggest to revise the list of abbreviations and align with the updated version of the manuscript as some terms were deleted e.g. PMTCT | Thanks so much for your kind remarks.  Thanks for the recommendation. The result section has been shortened by reducing the quotes in the write up in the revised manuscript  Clarity has been added to the phase of the lockdown from the abstract up to the method’s section. In the abstract, this appears as “*The study sought to understand the experiences and perceptions of healthcare providers at the frontline during the first phase of the lockdown as they offer maternal and newborn health care services in both public and private health facilities in Uganda with the aim of streamlining patient care in face of the current COVID-19 pandemic and in future disasters*”.  In the introduction, further clarity on when the phase of the lockdown has been added. It now appears as “  *It’s against this background that the study sought to understand the lived experiences and perceptions of the health workers offering maternal and newborn services during the first phase of the lockdown to contain the COVID-19 pandemic during the COVID-19 pandemic in Uganda with the aim of streamlining patient care in the current and similar future disasters*”  Then in the method’s section, the write up has been changed to *“We conducted this embedded qualitative study as part of a bigger study that assessed the impact of COVID-19 pandemic on the provision of Maternal and Newborn healthcare services in eight health facilities in Kampala, Uganda between June 2020 and December 2020[30] during the first phase of the lockdown”.*  Thanks for the advice. The file name has been edited from “Raw data” to ”Detailed summary of the data by theme with quotes”  Thanks for the keen observation. The interview guide has been changed.  The sentence has been edited in the Abstract to “*With the travel restrictions, social distancing associated with the containment of theCOVID-19 pandemic, healthcare providers could be faced with challenges of accessing their work stations, and risk burn out as they offer maternal and newborn services*”.  Thanks for the advice. The write up has been changed to *“The interview guide primarily explored the lived experiences of healthcare providers as they offer maternal and newborn healthcare services during the COVID-19 pandemic*”.  The first sentence in the conclusion has been edited as advised by the reviewer to *“The COVID-19 Pandemic has led to a decline in quality of maternal and newborn service delivery by the healthcare providers as evidenced by shorter consultation time and failure to keep appointments to attend to patients…*”.  “U” has been written as “Uganda” in the edited manuscript. The sentence has also been shifted as recommended. The write up now appears as “*Uganda reported her first COVID-19 case on the 21st March 2020[4]. Since then the number of confirmed cases had reached 95,723 as of 06th August 2021 with 2,783 deaths reported by the Uganda Ministry of Health [5]. The COVID-19 pandemic took Uganda by surprise [6, 7]”.*  A reference has been added as recommended by the reviewer. It now appears as *“Interruption in access to quality maternal and newborn health services with the travel restrictions in place to curb the COVID-19 could put over 10,000 lives of both women and their babies in danger every single day of the COVID-19 pandemic [6]”.*  The manuscript has been revised accordingly to add information about the MNH situation in Uganda before the pandemic. It now appears as *“Prior to the COVID-19, Uganda through strategies like five year Health Sector Strategic Plans for the past two decades had reduced maternal mortality rates from 500 in 2000 to 375 deaths per 100,000 live births [22, 23]. The four visit antenatal attendance was at 59.9% from 33.1% in 2011 [24]. The unmet need for modern contraception had reduced to 26% from 30% in 2016[23, 25]. The fertility rate in Uganda stands at 4.3 currently from 5.3 in 2000 [22]. The postnatal care was still below optimal levels in Uganda at 54.3% [23]. The neonatal mortality was at 19.9 per 1000 live births before COVID-19 from 33 deaths per 1000 live births [22]. Neonatal tetanus protection had reached 85% as compared 52% in 2000 [26]. Significant progress had also been seen in the BCG immunization at 1 year with 88% while that of Haemophilus influenzae type B (Hib) and Diphtheria, Pertussis (whooping cough), and Tetanus (DPT) vaccine coverage was at 93% [23] before COVID-19 pandemic”.*  The background has been edited accordingly. The structure of the health system in Uganda is written out as *“Healthcare in Uganda is offered mainly by public (70%), private-Not for profit (20%) and private health facilities (10%) [9, 10]. Public health facilities are structured in the following categories; National and Regional referral hospitals, general hospitals, district hospitals/ Health centre IVs (offering care to a population of 100,000 both in and outpatient services and emergency surgeries), Health Centre III (serving a population of 20,000 at the sub county level offering mainly outpatient and maternity services), Health centre II (serving a population of 5,000 and being run by an enrolled midwife) and the Health centre I (linking the community to the health system and being run by the village health teams with or without formal training). Care in the public facilities is free [11]. Private-Not-for Profit health facilities are mainly faith based facilities that offer care at a subsidized cost. The private health facilities are run by individuals or institutions with no exact control on how care is billed [12].*  The sentence was meant to mention that 15 healthcare providers had died of covid-19 as of 31^st^ December 2020. The revised manuscript now has even more updated figures. It appears as “*As of 7th July 2021, 37 Ugandan health workers had died of COVID-19[30]”.*  As suggested by other reviewers, the identity of the health facilities has been concealed.  Table 1 has been edited accordingly as recommended by the reviewer. More details have been added.  At the time of data collection, none of the selected health facilities were designed to treat COVID-19 patients. It’s the reason we didn’t capture this information in regards to admission of women with COVID-19 in our study. The ministry of Health had designated 2 facilities to manage the COVID-19 patients.  Thanks for the advice. The manuscript has been edited accordingly as advised by the reviewer. It now appears as “*This study was conducted in eight health facilities (two Private hospitals, three Private-Not-for Profit hospitals and three Public health facilities) in Kampala, Uganda. These eight facilities were purposively selected because they are the biggest service providers in the three sectors (public, private-not-for profit and private) offering maternal and newborn health care in Kampala”.*  The manuscript has been edited accordingly. It now appears as “*Disinfection protocols were observed prior to the interviews*.  The manuscript has been revised accordingly to highlight the sites for the in depth interviews. This now appears as *“All the in depth interviews were administered in English, the official language used in Uganda in quiet rooms at the different selected health facilities as recommended by the hospital administrators*”.  “Potential clients” has been rephrased as “potential participants” as recommended by the reviewer.  Correction has been made in the revised manuscript. It now appears as “*After ascertaining data saturation with no new emerging themes, we stopped the data collection [37, 38].*  Clarity has been added in the edited manuscript. It now appears as “*A pilot study was carried out with four healthcare providers to pretest and modify the interview guide. Data from the pilot study was also included in the analysis as the healthcare providers in the pilot were not included in the main study*”.  The edited manuscript has more clarity added in. It’s now written as “*Data was backed up on hard drives, online databases and two computers*”.  “Baseline” has been removed from the title. It now appears as *“Characteristics of participants”*  Table 2 has been formatted and resized. “Obstetrician/Gynecologist” has been used instead of “medical doctor” as recommended by the reviewer.  Table 2 has been edited as recommended by the review.  Since the interviews are implied in the table 2 by the number of participants at each facility, for clarity in the result’s section, we have added the text “. *Of the 25 interviews, six were in private; ten were in public, while nine were in private not-for profit health facilities”.*  Thanks for the advice, though in the prior reviewer’s comments, this was the suggestion. I have deleted the column on the page numbers as this might change with publication. I’ll keep reference however for the raw data as advised.  Clarity has been added in the revised manuscript to address this concern. It now appears as *“The following themes and subthemes were generated from the data analysis. Data comparisons during rigor analysis showed a number of similar experiences in maternal and newborn health service delivery irrespective of the health facility. There were however some disparities in the experiences within the different cadres. Nurses tended to use more of the public means when compared to the obstetricians/gynaecologists and administrators. The way the nurses, navigated through the hassle of transport to the workstations were different from the obstetricians/gynaecologists (Table 3).*  Thanks for the keen observation; the manuscript has been edited to imply that the patient load was high for the available workforce as some of the healthcare providers couldn’t access their work stations during the pandemic. The sub heading has been changed to “*High Patient turnover per the available workforce and Burnout*”  “Significant” has been deleted from the revised manuscript as advised by the reviewer”  The exclamation mark has been removed as advised by the reviewer.  Thanks for the observation; “COVID-19” has been adopted throughout the manuscript.  CME has been spelt out as advised by the reviewer.  The implied services have been added in the revised manuscript. It now appears as “*As highlighted by the discussants, there’s a need for the government to mediate a friendly working environment between the security personnel and the medical fraternity to minimize on delays seen during the pandemic that could have grossly affected maternal and newborn service delivery like immunization, health facility deliveries, antenatal and postnatal clinic attendance [11-13, 68]”.*  Thanks so much for advice but since it was recommendation from the healthcare providers, it would be respectful to have their views represented in the publication. More reading around this area will however be ensured.  The abbreviation list has been updated as advised by the reviewer. | Page 20-34  Page 1 Line 28-32  Page 6 Line 149-152  Page 7 Line 155-158  Page 1 Line 25-28  Page 2 Line 35-37  Page 2 Line 52-60  Page 4 Line 74-76  Page 6-7 Line 137-141  Page 6 Line 114-125  Page 4-5 Line 74-90  Page 7 Line 143  Page 8-14, Line 171,172  Page 8, Line 165-169  Page 13: Line 184-185  Page 13-14: Line 185-187  Page 14: Line 200  Page 14-15, Line 211-212  Page 15, Line 222-224  Page 15, Line 231  Page 17, Line 275  Page 18  Page 18  Page 17, Line 277-279  Page 18-22, Line 290-292  Page 18, Line 283-289  Page 19  Page 22, Line 299, Page 23, line 326, Page 24, Line 362  Page 22, Line 303  Page 30, Line 518  Page 35, Line 647-652  Page 38-39, Line 739-752 |
